# An Ethnobotanical Study of Traditional Knowledge and Uses of Medicinal Wild Plants among the Marakwet Community in Kenya

**DOI:** 10.1155/2020/3208634

**Published:** 2020-03-30

**Authors:** Bernard K. Wanjohi, Vincent Sudoi, Elizabeth W. Njenga, Wilson K. Kipkore

**Affiliations:** ^1^Department of Wildlife Management, University of Eldoret, P.O. Box 1125-30100, Eldoret, Kenya; ^2^School of Environmental Studies, Department of Environmental Biology, University of Eldoret, P.O. Box 1125-30100, Eldoret, Kenya; ^3^Department of Biological Sciences, University of Eldoret, P.O. Box 1125-30100, Eldoret, Kenya; ^4^Department of Forestry and Wood Sciences, University of Eldoret, P.O. Box 1125-30100, Eldoret, Kenya

## Abstract

Traditional plant knowledge and uses of medicinal wild plants were investigated among the Marakwet community in Kenya. Data were collected through interviews with seven traditional healers and 157 questionnaires for local community members. Traditional names of the plants by traditional healers and local community members were prepared as a checklist. Loss of traditional medicinal names of plants was ascertained with up to 60% overlapping in their nomenclature. The traditional medicinal plants treated 41 diseases within the region, of which local community members understood common ones for treating stomachache (94.8%), diarrhea (70.7%), chest problems (65.5%), and typhoid (63.8%). It was also clear that there was low knowledge index of medicinal plants by the local community members (23.6%) based on knowledge of traditional healers. Clearly, medicinal plants for treatment of malaria, diabetes, tetanus, and pneumonia were recognized by over 40% of the local community members, while plants treating arteriosclerosis, meningitis, arthritis, trachoma, smallpox, rheumatic fever, and gout were known by less than 10% of the respondents. Among plants, the use of roots for treatment was known by over 67% of the local community members compared to fruits, bark, bulb, and flowers (<10%). This low traditional medicinal knowledge in a community relies on the traditional medicinal plants, calling for an urgent need to document the information and perpetuate this knowledge from one generation to another. This can be achieved by collecting the information and developing a database of medicinal plants for future research and potential development of new drugs.

## 1. Introduction

The use of indigenous plants in human medicine is well documented [1]. Current knowledge on medicinal plants as a source for relief from illness dates back to the early civilization in China, India, and the Near East [2–5]. Ingredients provided by plants have a wide range of medicinal properties [6–9]. Globally, about 60–80% of the people rely on herbal medicine as for primary healthcare needs [10–12]. Subsequently, the number of plants being recommended for use as herbal medicines has increased [13, 14]. In areas where there is perceived high cost of medical care, especially in Asia and Africa, medicinal plants have gained more recognition [15–18]. This stems from the affordability and accessibility of traditional medicine as a source of treatment in the primary healthcare system of resource-poor communities [19–21]. Therefore, focus on the knowledge of plants used in herbal medicines has been increasing.

It is now clear that knowledge of medicinal plants use as was embedded in indigenous cultures has slowly been eroding with modernization. Thus, over the years, the decline in cultural diversity has witnessed the erosion of human knowledge on medicinal plant species, their distribution, management, and methods of extracting the useful properties of medicinal plants [22]. Knowledge of the use of medicinal plants was derived mainly through traditional scholarly written traditional documentation of knowledge and pharmacopoeias for doctors and institutions, as well as Traditional Medical Knowledge (TMK), among households, communities, and/or ethnic groups. Rather than legislation and/or regulation, it has been suggested that suitable strategies to enhance sustainable utilization and management of medicinal plants are focusing on local approaches involving traditional medicinal knowledge [23, 24].

Most emphasis on the respect and perpetuation of knowledge about the medicinal plants is espoused by traditional medicinal knowledge (TMK). Although there are numerous reports, published work, thesis, dissertations, books, inventories, media reports, and monographs of the diversity of medicinal plants within the tropical environment [25–30], most of these knowledge are still based purely on scientific work that totally excludes the contribution of the local community members and does not reflect TMK. Of interest is that the majority of the works so far carried out in developing countries largely focus on the inventories, utilization, and conservation of medicinal plants [21, 30–35]. Various sets of recommendations have been compiled relating to the conservation of medicinal plants, such as those associated with international conferences at Chiang Mai, Thailand, in 1988 and Bangalore, India, in 1998 (http://www.frlht-india.org). Regardless, there is little application of TMK on these inventories.

There is enormous knowledge on the use of indigenous medicinal plants in Kenya over the last decades (e.g., [30, 32, 36–42]). In light of this, therefore, there is a high expectation of enormous traditional knowledge of medicinal plant species in Kenya due to the use of diverse plant species, diversity of cultures, diverse languages, and beliefs among the different ethnic groups in Kenya. To our knowledge, there are no data regarding the traditional medicinal plant knowledge and use by several local communities in Kenya. Moreover, Kenya is one of the countries experiencing dynamic changes in cultural norms and system, which renders the traditional and local knowledge of medicinal plants to be easily forgettable as most of the indigenous traditional knowledge is transferred to the local community members orally. Therefore, the current study was conducted to assess and document the traditional and local knowledge of medicinal plants and use among traditional healers and local community members in Marakwet, Kenya.

## 2. Materials and Methods

### 2.1. Study Area

**Figure 1:**
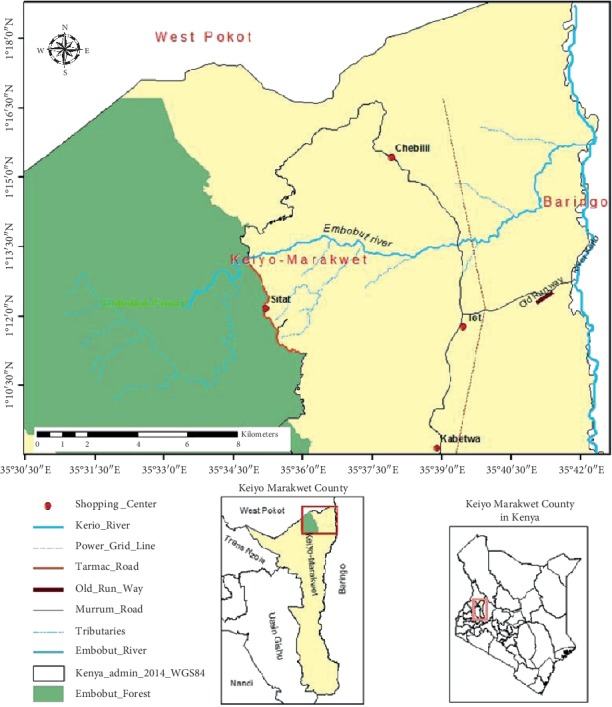
Map showing the location of the study area and sampling points.

This study was conducted at four sites within the Embobut Region of Elgeyo Marakwet County (Kenya) between March and June 2018 (Figure 1). Embobut covers an area of 21,655 hectares. The mean annual rainfall ranges between 1100 and 1500 mm with two peaks in April to May and August to October [43]. Temperature ranges between 14°C and 25°C with an average of 21°C. Soils are thin, drain freely, and have a friable texture with layers of cellular iron stone. It is a water catchment area for River Nzoia flowing into Lake Victoria. The main human activities within the study areas include livestock grazing, pastoralism, and crop and dairy farming. The main farming activities within the drainage areas include cultivation of maize (*Zea mays*), beans (*Phaseolus vulgaris*), and a variety of other noncereal crops on a smaller scale, including cabbage (*Brassica oleracea* var *capitata*) and kales (*Brassica oleracea* var *acephala*).

### 2.2. Population, Sample Size, and Sampling

The population was approximately 26,772 based on the KNBS (2010). From this population, about 3123 people (11.6%) had access to the forest [43]. The sample size was determined by the formula: *n*=*z*^2^(*pq*/*d*^2^) [44], whereby *n* = the desired minimum sample size, *z* = the standard normal deviation at set confidence interval, *d* = the acceptable range of error (0.05), *p* = the proportion of individuals accessing the forest (11.6%), and *q* = the proportion of individuals not accessing the forest = 1 − *p* (88.4%). Hence, *d* = 0.05, *p* = 0.116, *z* = 1.96 at 95% confidence level, and *q* = 0.884. Thus, *n*=1.96^2^((0.116*∗*0.884)/0.05^2^)=157. Therefore, the desired sample size was 157 local community members from the homesteads.

A purposive was used to select the 157 respondents. In purposive sampling, the participants are selected on the basis of some specific criteria that are judged to be essential [45]. The researcher deliberately selected community members with a long period of resident in the community, which signify knowledge of the natural environment and the use of natural resources to fulfill basic needs.

### 2.3. Research Instruments

Data were collected through traditional healers' survey interviews and local community questionnaires. Information on identification and use of local plant species was conducted through a census with seven traditional healers who were present during the time of study. The interviews were facilitated by translators who were well-conversant of the local language (Marakwet language). Local medicinal knowledge of plants use was obtained from questionnaires administered to the local community members. The questionnaires were designed to answer the following research questions: (1) which medicinal plant species do you know in the wild? (2) what are the plants used for? and (3) which plant parts are used? The names of the plants known by the traditional healers and local community members were noted in a checklist containing the vernacular and common names and submitted to the National Museum of Kenya (NMK) for confirmation by a plant taxonomist.

### 2.4. Piloting

A reconnaissance visit was conducted for four days to gain basic understanding of the potential respondents for the study. After the initial visit, a week was spent preparing interviews and questionnaires for the survey and another week for training of research assistants on how to effectively administer the instruments. The services of a translator were employed where necessary. A total of 24 questionnaires were piloted. The results of the pilot were used to improve efficiency of the data collection instruments for the main survey.

### 2.5. Validity and Reliability of Research Instruments

To test the validity of the research instruments, a questionnaire was prepared and submitted to ethnobotany researchers for cross checking and also to assess the reliance of the content.

Reliability of the research instruments was performed during pilot through the split half technique and Cronbach alpha coefficient computed [46]. Here, the instruments were provided to a total of 24 household heads divided into 2 groups. The reliability of the items was based on the estimates of the variability of responses between the two groups. In this study, the reliability coefficient was found to be 0.85, which was very good for the analysis.

### 2.6. Data Analysis

Quantitative data were cleaned, coded, and entered into Statistical Package for Social Science (SPSS) version 23 for analysis. Descriptive and cross tabulations were carried out. On the other hand, qualitative data were analyzed through synthesized text summaries and frequency distributions.

### 2.7. Ethical Considerations

This study adhered to the Ethical Standards of the University of Eldoret. Informed consent was sought and obtained before the study. Anonymity was ensured by not collecting identifying information of individual subjects. Confidentiality was ensured by not divulging the identity of the respondents or their organizations.

## 3. Results

### 3.1. Socioeconomic Background of the Respondents

The socioeconomic profile of the respondents is provided in Table 1. There was gender disparity in the villages with higher number of males (71%) than females (29%). Most of the respondents were aged over 55 years (39%) following those aged 46–55 years (29%), while those aged below 25 year were few. In Embobut, most household heads had no formal education (38%), which was followed by those with secondary levels of education (32%) and then primary level of education (27%). Generally, most farmers in the study area were practicing mixed farming (58%) followed by informal employment (16.2%). The majority of the households had stayed in the area for over 30 years (56.9%), followed by those who have stayed in the area for 10–19 (22.4%), while those who have stayed in the region for less than 10 years were few in proportion.

### 3.2. Traditional Medicinal Knowledge and Use of Indigenous Medicinal Plant Species in Embobut Forest

Structured interviews with the traditional healers of the local community documented 115 indigenous medicinal plant species (Table 2; Supplementary [Supplementary-material supplementary-material-1]). There was loss of traditional medicinal names of the plants with up to 69 indigenous plants (60%) overlapping in their naming. Meanwhile, up to 53 plant species (45.3%) had overlap in their names resulting in two names for a single plant species with another 14 (12.2%) having an overlap of three traditional names for a local plant. The number of plant species that had a single and consensus name among the traditional healers was 46 (40%). The local community members also identified the plant species based on the scheme developed by the traditional healers. The average identification index of the species among the locals was only 37.8%. Only 3 local community members (2.6%) positively identified all the medicinal plants. A total of 13.8% of the local community members were able to identify over 75% of the medicinal plants, while another 57.8% positively identified at least 50% of the medicinal plants. Meanwhile, up to 13% of the local community members could only identify less than 20% of the plant species.

**Figure 2:**
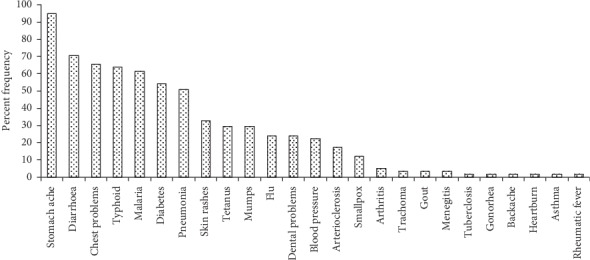
Local knowledge of the types of diseases within the Embobut Forest region (*n* = 116).

The study established that the traditional healers had wide knowledge of the local diseases with an identification of 41 diseases occurring within the region. The knowledge of the local community members concerning the types of diseases within the Embobut Forest region is shown in Figure 2. The local identified 25 diseases within the region and disease that are known by majority of the local community members were stomachache (94.8%), diarrhea (70.7%), chest problems (65.5%), and typhoid (63.8%). Other diseases such as malaria, diabetes, and pneumonia were identified by 61.2%, 54.3%, and 51.2% of the local community members, respectively.

The local knowledge of the numbers of indigenous medicinal plants used in management of diseases is shown in Table 3. The overall mean knowledge index of the number of medicinal plant species used in treatment of various diseases indicates a knowledge index of 23.6%. The knowledge of the use of local trees for treatment of malaria, diabetes, tetanus, and pneumonia was fairly known by the majority of the local community members (>40%) while plants for the treatment of arteriosclerosis, meningitis, arthritis, trachoma, smallpox, rheumatic fever, and gout were known by less than 10% of the respondents.

Information on the local medicinal knowledge of the indigenous plant parts used for treatment of diseases in the Embobut Forest region is shown in Table 4. Based on the data, the use of roots as a medicinal part of the plant known by 67% of the local community respondents. On the other hand, the use of stems, branches, and leaves in management of disease was understood by between 20 and 50% of the respondents. The knowledge of the use of the remaining parts of the plants, viz, fruits, bark, bulb, and flowers were known by less than 10% of the local community members.

## 4. Discussion

In the current study, a total of 115 medicinal plant species in 27 families were used by traditional healers in the Marakwet ethnic community of Kenya. A previous study in the same region yielded a total of 111 medicinal plants used by the local community [39]. Knowledge of the number of medicinal plants in the current study was higher than that in Samburu area [47] as well as in Baringo County [48]. Based on the documented medicinal plant used, it is suggested that there may be more medicinal plants used by the Marakwet or the knowledge of the traditional plants being used for medicinal purpose is much better than in other areas or both. However, during the study, there was also evidence of possible loss of traditional medicinal knowledge as attested by the traditional healers who expressed diverse knowledge on naming of medicinal plants. Indeed, the study established an overlap in naming of up to 60% of the medicinal plants by traditional healers, where 1.7% of the identified species had up to 4-5 names that overlapped in their local nomenclature while up to 45.3% overlapped in their names and 12.2% overlapped with upto three traditional names. Only 40% of the species had a single and consensus name among the traditional healers. Overlapping of traditional names of trees is one way that has been established to result in the loss of traditional medicinal knowledge [49–53]. It is possible that loss of traditional medicinal knowledge may be attributed to the nature of transmission of traditional medicinal knowledge from one generation to the other, which has often been orally performed.

During the study, the computed medicinal plant identification index was 37% indicating that out of every 100 plant species, the locals managed to identify positively only 37. It was even more surprising that only 2.6% of the local community members managed to identify all the medicinal plants, while up to 13% of the local community members could only identify less than 20% of the plant species. These results suggest an erosion of traditional medicinal knowledge, which concurs with other studies elsewhere [54, 55].

Stomach ache, malaria, diarrhea, chest problems, and typhoid (63.8%) were more prevalent diseases identified by the local community members. Other diseases such as malaria, diabetes, and pneumonia were identified by 61.2%, 54.3%, and 51.2% of the local community members, respectively. The knowledge index of medicinal plant species for treating various diseases was low among the local community members. The knowledge of medicine plants use was largely associated with common diseases in the area. However, the plants for the treatment of arteriosclerosis, meningitis, arthritis, trachoma, smallpox, rheumatic fever, and gout which are rare in the region were known only by the traditional healers and few local community members.

The study also established low levels of traditional knowledge of medicinal plant parts used. While knowledge of the use of root was wide among the local community members, the knowledge of the use of stem, branches, and leaf in management of disease was low among the local community members as well as the knowledge of the use of fruits, bark, bulb, and flowers. The discrepancies between knowledge and use indicate a possible erosion of local knowledge.

## 5. Conclusions

This study established that the traditional medicinal knowledge of medicinal plant use among Marakwets in Kenya was low or facing erosion. There is, therefore, an urgent need to document this information, as it is rapidly disappearing due to influence of western medicine and other reasons including sociocultural issues and overexploitation coupled with rapid deforestation. It is important to collect this information and develop a database of medicinal plants for future research and potential development of new drugs.

## Figures and Tables

**Table 1:** Respondents' socioeconomic and demographic characteristics.

	Characteristics	Frequency	Percent
Gender	Male	82	70.7
	Female	34	29.3
	Total	116	100

Age	<25	1	0.9
	26–35	11	9.6
	36–45	26	22.6
	46–55	33	28.7
	Above 55	45	39.1
	Total	116	100

Education level	None	44	37.9
	Primary	31	26.7
	Secondary	37	31.9
	College	4	3.4
	Total	116	100

Occupation	Crop farming	16	13.7
	Herder (animals)	4	3.4
	Mixed farming	68	58.1
	Traditional herbalist	2	1.7
	Formal employed	3	2.6
	Business	3	2.6
	Technicians	2	1.7
	Informally employed	19	16.2
	Total	117	100

Residence	Endo sibou	30	25.9
	Endo kibriem	30	25.9
	Embobut	30	25.9
	Kapiego	26	22.4
	Total	116	100.0

Duration of stay (years)	<10	3	2.6
	10–19	26	22.4
	20–29	21	18.1
	>30	66	56.9
	Total	116	100.0

**Table 2:** Local identification of plant species in Embobut Forest based on TMK (*n* = 116).

Attributes	Frequency	Percent
Number of medicinal species identified through TMK	115	—
Number of local community respondents	116	—
Number of medicinal species with 4 or 5 overlapping local names among traditional healers	2	1.7
Number of medicinal species with 3 overlapping local names among traditional healers	14	12.2
Number of medicinal species with 2 overlapping local names among traditional healers	53	46.0
Number of medicinal species without overlapping local names among traditional healers	46	40.0
Mean TMK knowledge index (%)	37.1	37.8
Members of local community who identified 100% of medicinal use	3	2.6
Members of local community who identified ≥75% of medicinal use	16	13.8
Members of local community who identified ≥50% of medicinal use	67	57.8
Members of local community who identified ≤20% of medicinal use	15	12.9

**Table 3:** Local knowledge of the numbers of indigenous plants used in management of diseases using the indigenous plant species (*n* = 116).

	Number of plants used (traditional healers)	Weighted knowledge of use index (%)
Disease managed		Frequency
Stomach ache	44	33.5
Diarrhea	46	24.5
Chest problems	12	32.1
Typhoid	29	22.8
Malaria	35	76.4
Diabetes	25	61.2
Pneumonia	13	44.5
Skin rashes	19	34.5
Tetanus	19	45.6
Mumps	18	43.6
Flu	16	32.4
Dental problems	16	23.5
Blood pressure	15	12.3
Arteriosclerosis	15	9.3
Smallpox	11	3.4
Arthritis	13	4.7
Trachoma	9	3.4
Gout	5	2.3
Meningitis	5	4.8
Tuberculosis	3	14.5
Gonorrhea	7	12.5
Backache	3	14.4
Heartburn	10	20.3
Asthma	8	11.4
Rheumatic fever	9	2.4
Average		23.6

**Table 4:** Local knowledge of the indigenous medicinal plant parts used for treatment of diseases in the Embobut Forest region (*n* = 116).

Plant parts used	Number of plants parts used (traditional healers)	Weighted local knowledge of plant parts used (%)
Roots	42	67.2
Stem	29	48.6
Branches	8	43.7
Leaf	12	21.4
Fruit	9	8.3
Bark	7	6.6
Bulb	4	5.7
Flowers	5	6.9

## Data Availability

The data used to support the findings of this study are available from the corresponding author upon request.

## Supplementary Materials

Supplementary Materials Supplementary Table 1. Local identification, scientific name, common names, and families of the identified medicinal plant species in Embobut Forest based on local traditional knowledge (*n* = 116).
