# Supplementary material for: An Ethnobotanical Study of Traditional Knowledge and Uses of Medicinal Wild Plants among the Marakwet Community in Kenya
Source: Evid Based Complement Alternat Med. 2020 Mar 30;2020:3208634. doi: 10.1155/2020/3208634 (PMC7149339; doi:10.1155/2020/3208634)
Supplement: Supplementary Materials — Supplementary Table 1. Local identification, scientific name, common names, and families of the identified medicinal plant species in Embobut Forest based on local traditional knowledge (n = 116). [file 3208634.f1.doc]

Supplementary Table 1. Local identification, scientific name, common names and families of the identified medicinal plant species in Embobut Forest based on local traditional knowledge (n = 116)

| **Local Name checklist** | **Scientific_Name** | **Voucher Number** | **Common name** | **Family** | **Medicinal use** | **% knowledge** |
| --- | --- | --- | --- | --- | --- | --- |
| Tilak | *Acacia lahai* Steud. & Hochst ex Benth.  . | WBK/7/16/293 | Red thorn | Fabaceae | Stomach-ache (Wiito mo) | 37.9 |
| Lugumwo/Tegelde/Tegilde | *Acanthus eminens* C.B.Cl. | WBK/7/16/001 | Bear's breeches | Acanthaceae | Typhoid (Kibarus kot), Amoebiasis (Kibei), head-ache (Amo met), heart-problem (Amo mugulei) (Amo mugulei) | 31.0 |
| Kipsirim | *Achyranthes aspera* L. | WBK/7/16/021 | Devil's horsewhip | Amaranthaceae | Coughing (Rolyo) | 13.8 |
| Kiputkut/Kibutkut | *Acmella caulirhiza* Del. | WBK/7/16/094 | Toothache plant | Asteraceae | Teeth-problems (Amo kelat), Gingivitis (unknown) (unknown), bad-smell (Ngútai kat) | 31.0 |
| Sait/Sayit/Morowo | *Afrocrania volkensii* (Harms.) Hutch. | WBK/7/16/230 | Dogwood | Cornaceae | Typhoid (Kibarus kot), Amoebiasis (Kibei), stomach-ache (Wiita ma), vomiting (Ong'unye) | 68.1 |
| Kitong’wo/Kitang’wa | *Albizia anthelmintica* (A.Rich) Brongn. | WBK/7/16/295 | Goatweed | Fabaceae | Malaria (Essio), stomach-ache (Wiita ma) | 51.7 |
| Chalbat/Chalpat | *Aloe tweedieae* (Christian) Wabuyele | WBK/7/16/088 | Chinese aloe | Asparagaceae | Amoebiasis (Kibei), stomach-ache Wiita ma), rashes (Sasar), deworming (Kericheb sorit) | 37.9 |
| Pangani | *Amaranthus spinosus* L. | WBK/7/16/027 | Spiny pigweed | Amaranthaceae | Anti-venom (Kericheb ket), wound (Kimwaa) | 20.7 |
| Sesimwa/Sesimua | *Artemisia afra* Jacq | WBK/7/16/097 | African wormwood | Asteraceae | Malaria (Essio) | 10.3 |
| Malut/Maltwo/Kipsowor | *Asparagus falcatus*Thunb. | WBK/7/16/077 | Sicklethorn | Asparagaceae | Teeth-problem (Amo kelat), rashes (Sasar) | 17.2 |
| Kiraita | *Basella alba* L. | WBK/7/16/166 | Vinespinash | Basellaceae | Amoebiasis (Kibei), stomach-ache (Wiita ma) | 41.4 |
| Kipsolwen/Kipsoroin/Kipsuruny | *Berberis holstii* Engl. | WBK/7/16/168 | Barberry | Berberidaceae | Deworming, Stomach-ache (Wiita ma), pain relief | 55.2 |
| Katabut | *Berkheya spekeana* Oliv. | WBK/7/16/099 | Buffalo-tongue | Asteraceae | Amoebiasis (Kibei) | 10.3 |
| Chepkondiwo/Cheposiwach/Kreilis Jepkondewo/Jepkondewo | *Bidens pilosa* L. | WBK/7/16/101 | Blackjack | Asteraceae | Rashes (Sasar) | 20.7 |
| Konuch/Konuk | *Biophytum abyssinicum* Steud ex A.Rich | WBK/7/16/448 |  | Oxalidaceae | Rashes (Sasar), smallpox (Sasar) | 10.3 |
| Sorukwo/Serekwo | *Boscia coriacea* Graells | WBK/7/16/193 |  | Capparaceae | Stomach-ache (Wiito mo) | 10.3 |
| Ararat/Kibou | *Calotropis procera* (Aiton) W.T. Aiton | WBK/7/16/073 | Rubber bush | Apocynaceae | Vomiting (Ong'unye), diarrhea (Kipkaywai) | 10.3 |
| Cheptuiya/Komolwo | *Canthium schimperianum* A. Rich. | WBK/7/16/562 |  | Rubiaceae | Diarrhea (Kipkaywai), wound (Kimwaa) | 10.3 |
| Legatetwa | *Carrisa edulis (*Forssk.) Vahl. | WBK/7/16/057 | Simple-spined num-num | Apocynaceae | Stomach-ache (Wiita ma), amoebiasis (Kibei) | 37.9 |
| Montrich | *Chenopodium ambrosioides* L | WBK/7/16/210 | Mexican tea | Amaranthaceae | Deworming (Kericheb sorit) | 13.8 |
| Montrich | *Chenopodium opulifolium* Schrad ex W.D.J. Koch & *Ziz* | WBK/7/16/212 | Grey goosefoot | Amaranmthaceae | Coughing (Rolyo) | 10.3 |
| Busungwo/Pisingwo/Pising | *Clematis simensis* Fresen. | WBK/7/16/539 | Pine hyacinth | Ranunculaceae | Rashes (Sasar), head-ache (Amo met), meningitis | 82.8 |
| Sachan/Sakarta | *Cleome gynandra* L. | WBK/7/16/198 | Stinkweed/Spiderwisp | Cleomaceae | Stomach-ache (Wiita ma), Typhoid (Kibarus kot) | 48.3 |
| Torokwo-ngwony/Kibararia | *Clinopodium abyssinicum* (Benth.) Kuntze. | WBK/7/16/358 | Basilweed | Lamiaceae | Coughing (Rolyo) | 17.2 |
| Kioswa/Sitab oin/Chekelel | *Clutia abyssinica* Jaub & Spach. | WBK/7/16/271 | Large fruited lighting-bush | Euphorbiaceae | Teeth-problems (Amo kelat) | 10.3 |
| Kolowo | *Crateva adansonii* DC. | WBK/7/16/199 |  | Rubiaceae | Amoebiasis (Kibei) | 10.3 |
| Kimilta/Kimira | *Crotalaria polysperma* Kotschy | WBK/7/16/307 |  | Fabaceae | Vomiting (Ong'unye), diarrhea (Kipkaywai) | 17.2 |
| Kibichan | *Croton ciliatoglandulifer* Ortega | WBK/7/16/272 | Mexican croton | Euphorbiaceae | Stomach-ache (Wiita ma) | 20.7 |
| Taposwo/Taboswa | *Croton macrostachyus* Hochst. ex Delile. | WBK/7/16/274 | Broad-leaved croton | Euphorbiaceae | Stomach-ache (Wiita ma), wound, rashes | 93.1 |
| Jeleikta/Jeliita/Cheliite | *Cussonia spicata* Thunb | WBK/7/16/069 | Spiked cabbage tree | Araliaceae | Liver enlargement (Koetu koy) | 27.6 |
| Morkut | *Cyperus esculentus* L. | WBK/7/16/253 | Nutsedge | Cyperaceae | Eye problems (Amo konyon), typhoid (Kibarus kot), stomach-ache (Wiita ma), coughing (Rolyo), blindness (Korot), back-ache (Amobatai), head-ache (Amo met), chest problem (Amo takat), gingivitis (unknown), appetite loss (Mochomu kii), bad breadth (Ng’utio kot) | 55.2 |
| Kibungwach/Murutyo | *Cyphostemma cyphopetalum* (Fresen.)Desc. Ex Wild & *R.B. DrumDrum (Fresen.)Desc. Ex Wild & R. Drum* | WBK/7/16/641 |  | Vitaceae | Stomach-ache (Wiito mo), Teeth-problems (Amokelat), Gonorrhea (Unknown) | 37.9 |
| Korosion | *Dobera glabra* (Forssk.) Juss. ex Poir | WBK/7/16/591 |  | Salvadoraceae | Eye-problems (Amo konyin) , Amoebiasis (Kibei) | 10.3 |
| Tabilikwa/Taplikwo | *Dodonaea angustifolia* L.f. | WBK/7/16/597 | Sand olive/Hop bush | Sapindaceae | Rheumatic fever (Kimogonoi) | 20.7 |
| Borowo | *Dombeya torrida* (J.F. Gmel.)Bamps | WBK/7/16/400 | Forest dombeya | Malvaceae | Malaria (Essio), Stomach-ache (Wiito mo), vomiting (Ong'unye), goitre (Unknown) | 93.1 |
| Mindililwo/Mintrilwo | *Dovyalis abyssinica* (A. Rich.) Warb. | WBK/7/16/342 | Ceylon gooseberry | Flacourtiaceae | Stomach-ache (Wiito mo) (Wiita ma), aphrodisiac (Kerichek ab muren), aphrodisiac (Solit) | 27.6 |
| Lobchon/Turol | *Dryopteris inaequalis* (Schltdl.) Kuntz | WBK/7/16/260 |  | Polypodiaceae | Vomiting (Ong'unye), Mumps (Kipsabat) | 69.0 |
| Sagorgetia | *Englerina woodfordioides* (Schweinf.) Balle.. | WBK/7/16/394 | Short-barred sapphire | Loranthaceae | Liver enlargement (Koetu koy), heartburn (Amo mugulei) | 24.1 |
| Jeptekan | *Tapinanthus buvumae* (Rendle) Danser | WBK/7/16/395 |  | Loranthaceae | Heartburn (Amo mugulei) | 17.2 |
| Sosurwo/Sosurwa | *Ensete ventricosum* (Welw.) Cheesman | WBK/7/16/428 | Ethiopian banana | Musaceae | Liver enlargement (Koetu koy) | 10.3 |
| Jeptuiya/Uswo | *Euclea divinorum* Hiern. | WBK/7/16/263 | Towerghwarrie | Ebenaceae | Arthritis (Kimogonoi), leprosy (Unknown) | 17.2 |
| Kureswo/Kireswa | *Euphorbia candelabrum* Tremaux ex Kotschy | WBK/7/16/276 | Candelabra euphorbia | Euphorbiaceae | Back-ache (Amobatai), Mumps (Kipsabat), Blood pressur (Loyob Koroti) | 20.7 |
| Kokoja | *Faidherbia albida* (Delile) A.Chev. | WBK/7/16/311 | Apple-ring acacia, Winter thorn | Fabaceae | Liver enlargement (Koetu koy) | 24.1 |
| Sirirto/Maiyokwa/Markwa | *Faurea saligna* Harv. | WBK/7/16/534 | Beechwood | Proteaceae | Stomach-ache (Wiito mo) (Wiita ma), head-ache (Amo met) | 27.6 |
| Simotwo | *Ficus natalensis* Hochst. | WBK/7/16/425 | Back-cloth fig | Moraceae | Arthritis (Kimogonoi) | 17.2 |
| Poriotwo | *Ficus thonningii* Blume. | WBK/7/16/427 |  | Moraceae | Stomach-ache (Wiito mo) (Wiita ma) | 13.8 |
| Tingas/Tongururwo/Tungururwa | *Flacourtia indica* (Burm. f.) Merr. | WBK/7/16/343 | Governor’s plum | Flacourtiaceae | Anti-venom (Kericheb ket), Arthritis (Kimogonoi) | 31.0 |
| Nolkwo | *Garcinia livingstonei* T. Anderson. | WBK/7/16/213 | African mangosteen | Clusiaceae | Typhoid (Kibarusk kot), Stomach-ache (Wiito mo), Teeth-problems (Amokelat) | 41.4 |
| Mokilion | *Gardenia ternifolia* Schumach. & Thonn*.* | WBK/7/16/567 | Large-leaved Transvaalgardenia | Rubiaceae | Amoebiasis (Kibei), vomiting (Ong'unye) | 24.1 |
| Sewerwa/Soworwo | *Hagenia abyssinica* Willd. | WBK/7/16/556 | African redwood | Rosaceae | Stomach-ache (Wiito mo) (Wiita ma), allergy (Mokocho), gout (Unknown) | 48.3 |
| Kapkerelwa | *Harrisonia abyssinica* Oliv. | WBK/7/16/586 |  | Rutaceae | Typhoid (Kibarus kot), amoebiasis (Kibei), chest-problems (Amo takat), Stomach-ache (Wiito mo) (Wiita ma), Arthritis (Kimogonoi) | 58.6 |
| Tirgonio/Tirkonio | *Hypoestes forskaolii (*Vahl) R.Br. | WBK/7/16/012 | White ribbon bush | Acanthaceae | Stomach-ache (Wiito mo) (Wiita ma) | 10.3 |
| Kiptolion | *Indigofera arrecta* Hochst. ex A.Rich. | WBK/7/16/314 | Bengal Indigo | Fabaceae | Stomach-ache (Wiito mo) (Wiita ma), Teeth-problems (Amokelat) | 31.0 |
| Sarkelat/Sarkilat | *Indigofera atriceps* Hook.f. | WBK/7/16/315 |  | Fabaceae | Teeth-problems (Amokelat) | 10.3 |
| Kiptora/Kipkawa | *Jasminum abyssinica* N.E.Br. | WBK/7/16/439 |  | Oleaceae | Coughing (Rolyo), Aphrodisiac (Solit), Wound (Kimwaa) | 20.7 |
| Torokwo | *Juniperus procera* Hochst. ex Endl. | WBK/7/16/250 | African pencil cedar | Cupressaceae | Amoebiasis (Kibei), Stomach-ache (Wiito mo) (Wiita ma), Aphrodisiac (Kerichek ab muren) | 44.8 |
| Cheporus/Tirkonio | *Justicia flava* (Forsk.) Vahl | WBK/7/16/017 | Yellow justicea | Acanthaceae | Coughing (Rolyo), Vomiting (Ong'unye) | 13.8 |
| Kamuserwo | *Kalanchoe crenata* (Andrews) Haw. | WBK/7/16/234 | Neverdie | Crassulaceae | Rashes (Sasar) , Disinfectant | 34.5 |
| Rotio/Rotion | *Kigelia africana (*Lam.) Benth. | WBK/7/16/169 | Sausage tree | Bignoniaceae | Leprosy (Unknown), Rashes (Sasar) , aphrodisiac (Solit) | 13.8 |
| Loloito/Lolotwo | *Lannea fulva* (Engl.) Engl. | WBK/7/16/036 |  | Anacardiaceae | Head-ache (Amo met) | 17.2 |
| Morno | *Lannea schweinfurthii* (Engl.) Engl | WBK/7/16/037 | False marula | Asteraceae | Amoebiasis (Kibei), Stomach-ache (Wiito mo) (Wiita ma), waterborne diseases, Arthritis (Kimogonoi) | 41.4 |
| Sikiroi/Chururur | *Lonchocarpus eriocalyx* Harms. | WBK/7/16/317 |  | Fabaceae | Malaria (Essio), Stomach-ache (Wiito mo) (Wiita ma), Waterborne diseases (Unknown), Gonorrhea (Unknown) | 31.0 |
| Mborio/Ribotio | *Maerua crassifolia* Forssk. | WBK/7/16/532 | False Assegai | Capparaceae | Stomach-ache (Wiito mo) (Wiita ma) | 17.2 |
| Chepkingung | *Momordica anigosantha*Hook.f. | WBK/7/16/245 | Bitter Melon | Cucurbitaceae | Vomiting (Ong'unye) | 34.5 |
| Cheseria/Jeseria | *Momordica foetida* Schumach | WBK/7/16/246 | French concombre sauvage | Cucurbitaceae | Amoebiasis (Kibei), Chest-problems (Amo takat), Stomach-ache (Wiito mo) (Wiita ma), Teeth-problems (Amokelat), head-ache (Amo met) | 84.5 |
| Segatet | *Myrsine africana* L. | WBK/7/16/431 | African boxwood | Myrsinaceae | Typhoid (Kibarusk kot) | 17.2 |
| Chorwo | *Nuxia congesta R.Br. ex* Fresen. | WBK/7/16/624 | Brittlewood | Stilbaceae | Malaria (Essio), Typhoid (Kibarusk kot), smallpox (Sasar), Stomach-ache (Wiito mo) (Wiita ma), pneumonia, rashes (Sasar), varicose veins, vomiting (Ong'unye), Mumps (Kipsabat), tonsols, oedema | 99.1 |
| Remit/Yemit | *Olea europaea* L. | WBK/7/16/441 | Olive tree | Oleaceae | Typhoid (Kibarusk kot), Stomach-ache (Wiito mo) (Wiita ma), flu (Tung’ion), Coughing (Rolyo), heartburn (Amo mugulei), aphrodisiac (Solit) | 86.2 |
| Mutung'wa/Mutung'wo | *Ozoroa insignis* Delile | WBK/7/16/041 | Tar berry | Anacardiaceae | Stomach-ache (Wiito mo) (Wiita ma), head-ache (Amo met), cancer, vomiting (Ong'unye), heart-problem (Amo mugulei) | 82.8 |
| Kipchee | *Pergularia daemia (*Forssk.) Chiov. | WBK/7/16/062 | Trellis-vine | Apocynaceae | Stomach-ache (Wiito mo) (Wiita ma), rashes (Sasar) | 55.2 |
| Sinendo/Sinondo | *Periploca linearifolia* Quart.-Dill & A.Rich. | WBK/7/16/063 | Silk vine | Apocynaceae | Eye-problems (Amo konyin), amoebiasis (Kibeii), Stomach-ache (Wiito mo) (Wiita ma), Wound (Kimwaa) | 27.6 |
| Borio | *Peucedanum aculeolatum* Engl. | WBK/7/16/051 | Wild Parsley | Apocynaceae | Cancer (Koroibo him) | 10.3 |
| Chemnowo | *Pittosporum viridiflorum* Sims | WBK/7/16/459 | Cheesewood | Pittosporaceae | Head-ache (Amo met), vomiting (Ong'unye), bronchitis (Unknown), asthma (Unknown) | 58.6 |
| Ang'urwet/Ang’uur | *Plectranthus barbatus Andrews.* | WBK/7/16/377 | Indian coleus | Lamiaceae | Teeth-problems (Amokelat), tetanus (Unknown), disinfectant | 96.6 |
| Ben/Benet | *Podocarpus gracilior* Pilg. | WBK/7/16/519 | Weeping Podocarpus | Podocarpaceae | Stomach-ache (Wiito mo) (Wiita ma), Arthritis (Kimogonoi) | 79.3 |
| Tendwo/Tondwo//Tendwet | *Prunus africana* (Hook.f.) Kalkman | WBK/7/16/557 | Red stinkwood | Rosaceae | Typhoid (Kibarusk kot), Stomach-ache (Wiito mo) (Wiita ma), cancer, aphrodisiac (Kerichek ab muren) | 82.8 |
| Sitotwet/Karabar | *Rapanea melanophloeos* (L.) Mez | WBK/7/16/533 | Cape beech | Primulaceae | Head-ache (Amo met) | 31.0 |
| Kosisit/Kasisit | *Rhamnus prinoides* L. Her. | WBK/7/16/543 | African Dogwood | Rhamnaceae | Stomach-ache (Wiito mo) (Wiita ma), Gonorrhea (Unknown) | 37.9 |
| Sirian | *Rhus natalensis* Berhn*.* | WBK/7/16/546 | Natal rhus | Anacardiaceae | Stomach-ache (Wiito mo) (Wiita ma), Coughing (Rolyo), rashes (Sasar) , Liver enlargement (Koetu koy) | 44.8 |
| Monwo/Mania | *Ricinus communis* L. | WBK/7/16/287 | Castor-oil plant | Euphorbiaceae | Stomach-ache (Wiito mo) (Wiita ma) | 17.2 |
| Chebobet | *Rotheca myricoides* (Hochst.)Steane & Mabb. | WBK/7/16/385 | Butterfly Bush | Lamiaceae | Typhoid (Kibarusk kot), Stomach-ache (Wiito mo) (Wiita ma) | 31.0 |
| Momonwa | *Rubus steudneri* Schweinf. |  | Forest bramble | Rosaceae | Stomach-ache (Wiito mo) (Wiita ma) | 13.8 |
| Chekowo/Checha | *Salvadora persica* L. | WBK/7/16/592 | Toothbrush tree | Salvadoraceae | Stomach-ache (Wiito mo) (Wiita ma), Teeth-problems (Amokelat), Back-ache (Amobatai) | 31.0 |
| Tinwot/Tingwa/Tingwon | *Schefflera volkensii* (Harms) Harms | WBK/7/16/071 | Cabbage tree | Araliaceae | Malaria (Essio), Typhoid (Kibarusk kot), tuberculosis (Kipsosoi), pneumonia, goitre, rashes (Sasar), chest pains, allergies, Coughing (Rolyo), head-ache (Amo met) | 75.0 |
| Kipitkut | *Schkuhria pinnata* (Lam.) Kuntze ex Thell. | WBK/7/16/147 | Feathery false threadleaf | Asteraceae | Plague, gingivitis (unknown) | 17.2 |
| Tigagowa | *Scutia myrtina* (Burm. f.) Kurz | WBK/7/16/548 | Cat-thorn | Rhamnaceae | Stomach-ache (Wiito mo) (Wiita ma), allergies (Mokocho) | 37.9 |
| Korkor/Korkorio | *Sida cuneifolia R*oxb | WBK/7/16/412 | Common wireweed | Malvaceae | Typhoid (Kibarusk kot), flu (Tung’ion), Waterborne diseases (Unknown), head-ache (Amo met), Mumps (Kipsabat) | 79.3 |
| Lemeiwo/Lomoiwo | *Syzygium guineense* Wall. | WBK/7/16/435 | Water pear | Myrtaceae | Eye-problem, diarrhoea (Kipkaywai) | 20.7 |
| Labotwa/Jebokimnerkeny | *Solanum incanum* L. | WBK/7/16/615 | Sodom apple | Solanaceae | Stomach-ache (Wiito mo) (Wiita ma), Teeth-problems (Amokelat), Arthritis (Kimogonoi), Back-ache (Amobatai), liver-enlargement, Anti-venom (Kericheb ket) | 10.3 |
| Sikawa/Sikowo | *Solanum aculeastrum* Dunal | WBK/7/16/612 | Apple of Sodom | Solanaceae | Teeth-problem, Anti-venom (Kericheb ket) | 82.8 |
| Kalopotwo | *Solanum mauense* Bitter | WBK/7/16/616 |  | Solanaceae | Teeth-problems (Amokelat), flu (Tung’ion), Stomach-ache (Wiito mo) (Wiita ma) | 34.5 |
| Kisoyo/Kipongosi | *Solanum nigrum* L. | WBK/7/16/617 | Black nightshade | Solanaceae | Teeth-problems (Amokelat), Stomach-ache (Wiito mo) (Wiita ma) | 17.2 |
| Kisoyoborin | *Solanum terminale* Forssk. | WBK/7/16/620 |  | Solanaceae | Rashes (Sasar) | 75.9 |
| Oron | *Tamarindus indica* L | WBK/7/16/328 | Tamarind/Athel tree | Fabaceae | Typhoid (Kibarusk kot), flu (Tung’ion), Coughing (Rolyo) | 94.8 |
| Koloswo/Goloswa/Groswo | *Terminalia brownii* Fresen. | WBK/7/16/217 |  | Combretaceae | Malaria (Essio), cancer, blindness, Typhoid (Kibarusk kot), diabetes, Coughing (Rolyo), eye-problem, Stomach-ache (Wiito mo) (Wiita ma), Teeth-problems (Amokelat), Waterborne diseases (Unknown) | 98.3 |
| Kipkeres/Kipkutai | *Toddalia asiatica* (L.) Lam. | WBK/7/16/589 | Cockspur Orange | Rutaceae | Typhoid (Kibarusk kot), amoebiais (Kibei), Stomach-ache (Wiito mo) (Wiita ma), Back-ache (Amobatai), cancer, aphrodisiac (Kerichek ab muren) | 37.9 |
| Kilesan/Kreswo | *Tribulus terrestris L.* | WBK/7/16/645 | Caltrop | Zygophyllaceae | Arthritis (Kimogonoi) | 20.7 |
| Kimilei | *Urtica massaica* Mildbr. | WBK/7/16/633 | Maasai stinging nettle | Urticaceae | Vomiting (Ong'unye), leukemia (Kroibi korot) | 37.9 |
| Ngapko/Ngobgwa/Angapwo | *Vachellia nilotica* (L.) P.J.H. Hutler & Mabb. | WBK/7/16/333 | Scented thorn | Fabaceae | Liver-enlargement, chest-pains, diarrhea (Kipkaiywai) | 13.8 |
| Labeiywa/Chesamis | *Vachellia nubica* (Benth.) Kyal & Boatwr. | WBK/7/16/334 |  | Fabaceae | Typhoid (Kibarusk kot), amoebiasis (Kibeii) | 17.2 |
| Reno/Rena | *Vachellia seyal* (Delile) P.J.H. Hurter | WBK/7/16/336 | Red acacia | Fabaceae | Amoebiasis (Kibeii) (Kibei), Stomach-ache (Wiito mo) (Wiita ma), head-ache (Amo met) | 34.5 |
| Krorion/Kirorion | *Vernonia amygdalina* Delile | WBK/7/16/157 | Bitter leaf | Asteraceae | Typhoid (Kibarusk kot), amoebiasis (Kibeii) (Kibei), Stomach-ache (Wiito mo) (Wiita ma), Teeth-problems (Amokelat) | 51.7 |
| Ononion/Tabang'wa | *Vernonia auriculifera* Hiern | WBK/7/16/158 |  | Asteraceae | Eye-problem, Stomach-ache (Wiito mo) (Wiita ma), head-ache (Amo met), influ (Tung’ion)enza, kidney-stones | 87.1 |
| Sekwon/Sokwon/Sekwan | *Warburgia ugandensis* Sprague | WBK/7/16/191 | Ugandan greenheart | Canellaceae | Head-ache (Amo met), Teeth-problems (Amokelat) | 58.6 |
| Tarkukai/Kipkogai/Kwoleria | *Withania somnifera* (L.) Dunal | WBK/7/16/622 | Winter cherry | Solanaceae | Eye-problem, cancer (Koroibo him) | 34.5 |
| Tegan/Tegaa | *Yushania alpina* (K.Schum.) W.C. Lin | WBK/7/16/517 | Bamboo | Poaceae | Diarrhea, aphrodisiac (Solit) | 34.5 |
| Songoiywa/Songururwa | *Zanthoxylum chalybeum* Engl. | WBK/7/16/590 | Knot wood | Rutaceae | Typhoid (Kibarusk kot), amoebiasis (Kibeii) (Kibei), chest-problems (Amo takat), Stomach-ache (Wiito mo) (Wiita ma), Waterborne diseases (Unknown) | 89.7 |
| Cheserya/Kisangwa | *Zehneria scabra* (L.f.) Sond. | WBK/7/16/248 | Mouse melon | Cucurbitaceae | Rashes (Sasar) | 20.7 |
| Tilomwo/Tirak/Tilam | *Ziziphus mauritiana* Lam. | WBK/7/16/549 | Indian plum/Jujube | Rhamnacaeae | Amoebiasis (Kibeii) (Kibei), Stomach-ache (Wiito mo) (Wiita ma) | 81.0 |
